# Partnering for impact: Integrated transmission assessment surveys for lymphatic filariasis, soil transmitted helminths and malaria in Haiti

**DOI:** 10.1371/journal.pntd.0005387

**Published:** 2017-02-16

**Authors:** Alaine Kathryn Knipes, Jean Frantz Lemoine, Franck Monestime, Carl R. Fayette, Abdel N. Direny, Luccene Desir, Valery E. Beau de Rochars, Thomas G. Streit, Kristen Renneker, Brian K. Chu, Michelle A. Chang, Kimberly E. Mace, Kimberly Y. Won, Patrick J. Lammie

**Affiliations:** 1 Division of Parasitic Diseases and Malaria: U.S. Centers for Disease Control and Prevention; Atlanta, GA, United States of America; 2 Programme National de Malaria et de Filariose Lymphatique (PNCM), Ministère de la Santé Publique et de la Population (MSPP), Haiti; 3 IMA World Health, Washington, District of Columbia, United States of America; 4 University of Notre Dame, Léogane, Haiti; 5 Hôpital St. Croix, Léogane, Haiti; 6 Emerging Pathogens Institute, University of Florida, Gainesville, Florida, United States of America, Department of Health Services Research, Management, and Policy, College of Public Health and Health Professions, University of Florida, Gainesville, Florida, United States of America; 7 The Carter Center Atlanta, GA, United States of America; 8 Neglected Tropical Diseases Support Center, Task Force for Global Health, Decatur, GA, United States of America; Task Force for Global Health, UNITED STATES

## Abstract

**Background:**

Since 2001, Haiti’s National Program for the Elimination of Lymphatic Filariasis (NPELF) has worked to reduce the transmission of lymphatic filariasis (LF) through annual mass drug administration (MDA) with diethylcarbamazine and albendazole. The NPELF reached full national coverage with MDA for LF in 2012, and by 2014, a total of 14 evaluation units (48 communes) had met WHO eligibility criteria to conduct LF transmission assessment surveys (TAS) to determine whether prevalence had been reduced to below a threshold, such that transmission is assumed to be no longer sustainable. Haiti is also endemic for malaria and many communities suffer a high burden of soil transmitted helminths (STH). Heeding the call from WHO for integration of neglected tropical diseases (NTD) activities, Haiti’s NPELF worked with the national malaria control program (NMCP) and with partners to develop an integrated TAS (LF-STH-malaria) to include assessments for malaria and STH.

**Methodology/Principle findings:**

The aim of this study was to evaluate the feasibility of using TAS surveys for LF as a platform to collect information about STH and malaria. Between November 2014 and June 2015, TAS were conducted in 14 evaluation units (EUs) including 1 TAS (LF-only), 1 TAS-STH-malaria, and 12 TAS-malaria, with a total of 16,655 children tested for LF, 14,795 tested for malaria, and 298 tested for STH. In all, 12 of the 14 EUs passed the LF TAS, allowing the program to stop MDA for LF in 44 communes. The EU where children were also tested for STH will require annual school-based treatment with albendazole to maintain reduced STH levels. Finally, only 12 of 14,795 children tested positive for malaria by RDT in 38 communes.

**Conclusions/Significance:**

Haiti’s 2014–2015 Integrated TAS surveys provide evidence of the feasibility of using the LF TAS as a platform for integration of assessments for STH and or malaria.

## Author summary

Lymphatic filariasis and malaria are mosquito-borne parasitic infections that are endemic in Haiti. Soil-transmitted helminths are also present in Haiti, infecting large numbers of people every year. Since 2001, Haiti’s National Program for the Elimination of Lymphatic Filariasis (NPELF) has worked to reduce the transmission of LF through annual mass drug administration with the aim of reducing LF prevalence in the population below a threshold, such that transmission is assumed to be no longer sustainable. By treating the entire population of Haiti with a combination of drugs, the elimination program has made tremendous progress towards eliminating the disease. By 2014, Haiti’s NPELF had met the World Health Organization eligibility criteria to conduct LF transmission assessment surveys (TAS) and decided to use the LF TAS as a platform to collect information about STH and malaria. The WHO has called for the integration of program activities in the field, and the TAS is a platform that allows for such integration. In Haiti the integrated TAS reduced the burden of repeated surveys on communities by minimizing site visits and benefited all three disease programs by sharing the responsibilities of field data collection.

## Introduction

Globally, lymphatic filariasis (LF), soil transmitted helminths (STH) and malaria are frequently co-endemic, presenting opportunities for integration of programs targeting their control and elimination. The WHO Global Program to Eliminate LF (GPELF) was launched in 2000 with a commitment to the elimination of LF as a public health problem by 2020 through mass drug administration (MDA) [1]. By 2013, more than 4.4 billion treatments of diethylcarbamazine or ivermectin plus albendazole (DEC+ALB or IVM+ALB) had been distributed in 56 countries, achieving an estimated 46% reduction in the population at risk of LF from 1.46 billion to 789 million people [1,2,3]. In 2012, an estimated 1.5 billion people were infected with STH globally [4]. In 2014, an estimated 269 million pre-school-aged (PSAC) and 576.6 million school-aged children (SAC) were living in areas endemic for STH, which WHO recommends be addressed with periodic administration of ALB or mebendazole (MBZ) preventive chemotherapy (PC)[4]. Globally, the WHO target is to treat at least 75% of children living in STH endemic countries with PC by 2020 [5]. Approximately 3.2 billion people live in areas where they are at risk for malaria transmission [6]. The WHO global technical strategy for malaria (2016–2030) aims to ensure universal access to malaria prevention, diagnosis and treatment, to accelerate efforts towards elimination and attainment of malaria-free status, and to transform malaria surveillance into a core intervention [7]. Occurring in the tropical and subtropical zones, LF and malaria are both transmitted by mosquito vectors, and in certain areas, by the same species.

The island of Hispaniola is the only remaining Caribbean island that is endemic for both malaria and LF [8], with Haiti bearing the greater burden of both diseases. In 2001, Haiti’s National LF elimination program (NPELF) determined that nearly all communes were endemic for LF [9] and began administration of MDA (DEC + ALB) in select areas. Full national treatment coverage (140 communes) was achieved by 2012 [10]. In 2014, Haiti was one of the 25 countries in the Americas where PC was needed for STH, and one of seven in the region that achieved the ≥ 75% national coverage target [5]. Haiti’s malaria prevalence is low (0.4% in 2011) [11], though 17,662 confirmed cases were reported in 2014 [6]. Documented asymptomatic parasitemia [12, 13] highlights the need for surveillance strategies to identify remaining high transmission foci. To attain elimination of LF and malaria in Haiti, and control of STH, the three disease programs sought to identify remaining foci of disease transmission by integration of surveillance activities.

In 2011, the World Health Organization (WHO) recommended the Transmission Assessment Survey (TAS), a standardized and statistically rigorous survey for measuring LF prevalence [14]. WHO recommends that LF elimination programs conduct the TAS in areas that have: 1) received 5 or more effective annual rounds of MDA; and 2) where spot-check and sentinel site surveys indicate microfilaria (mf) prevalence is less than 1% or antigenemia prevalence is less than 2%. The WHO has called for integration of neglected tropical diseases (NTD) activities, and in 2015, released a protocol to integrate LF-TAS with an assessment for STH [15]. LF-TAS have successfully been integrated with STH assessments in various countries [16,17,18]. Results from these integrated surveys can be used to determine the frequency of school-based STH treatment needed after community-wide LF MDA stops. Haiti’s NPELF, National Malaria Control Program (NMCP) and partners saw an opportunity to synergize efforts for the LF and STH surveys in order to collect community-level information on malaria, as an integrated TAS for all three diseases (‘TAS-STH-malaria’). The decision for the LF and malaria programs to work together was facilitated by the fact that the both programs are led by the same director, and are housed within the same office at the Ministry of Public Health and Population (MSPP). To our knowledge, this was the first time the LF TAS was used as a platform for also assessing both STH and malaria. The aim of this study was to evaluate the feasibility of using TAS surveys for LF as a platform to collect information about STH and malaria.

## Methods

### Ethics statement

Transmission assessment surveys were conducted according to study protocols approved by institutional review board (IRB) of the United States Centers for Disease Control and Prevention and the Ethics Committee of the Haitian Ministry of Public Health and Population (MSPP). Following ministry policy, MSPP and IMA World Health staff recruited designated schools to participate in the survey and contacted the schools’ headmasters in advance to advise them of the purpose of the survey and to request that they notify parents. Children provided verbal assent at the time of the survey.

### Study site

**Fig 1:**
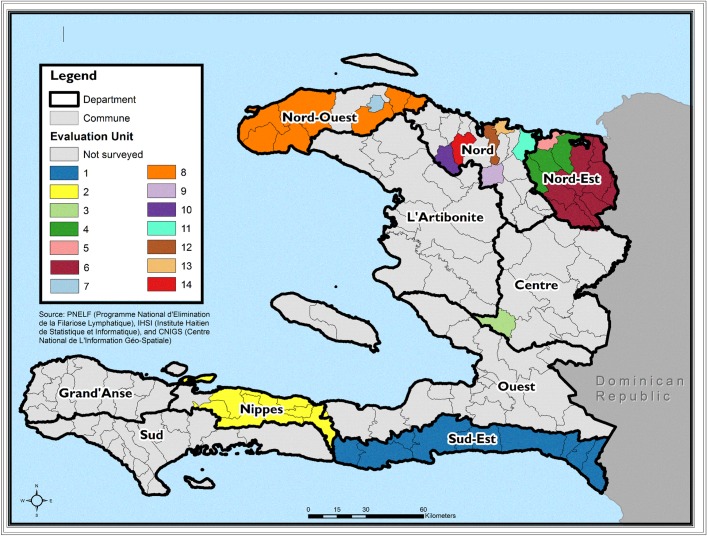
Integrated Transmission Assessment Surveys. Haiti 2014–2015.

At the time of the surveys, Haiti’s 10 departments were divided into 140 communes. Communes with the lowest baseline LF antigen prevalence based on the 2001 national survey were combined to form evaluation units (EUs); these EUs ranged from three or more communes to an entire department (maximum of 10 communes, Sud Est) (Table 1). Communes with greatest LF antigen prevalence at baseline were evaluated individually (n = 8 EUs). Altogether, surveys were conducted in 14 EUs, composed of 47 communes in 6 departments, where TAS eligibility requirements had been met (Fig 1). Between November 2014 and June 2015, TAS (LF-only) (EU 1), TAS-STH-malaria (EU 2) and TAS-malaria (EUs 3–14) were conducted. Due to the ambitious TAS schedule for 2014–2015, MSPP approved piloting of the three disease protocol (TAS-STH-Malaria) in one EU for 2015.

**Table 1:** Evaluation Units.

EU #	TAS type	Department	Number of communes in Department	Number of Communes per EU	Baseline LF antigen Prevalence (2001)
1	LF	Sud Est	10	10	0–4.9%
2	LF + malaria + STH	Nippes	11	10 ^A^	0–4.9%
3	LF + malaria	Centre	12	1 ^B^	0–4.9%
4	LF + malaria	Nord Est	13	2 ^C^	5–9.9%
5	LF + malaria	Nord Est	13	1 ^D^	10–45%
6	LF + malaria	Nord Est	13	9 ^E^	0–4.9%
7	LF + malaria	Nord Ouest	10	1 ^F^	7.0%
8	LF + malaria	Nord Ouest	10	7 ^G^	2.9%
9	LF + malaria	Nord	19	1 ^H^	14.0%
10	LF + malaria	Nord	19	1 ^I^	30.0%
11	LF + malaria	Nord	19	1 ^J^	37.4%
12	LF + malaria	Nord	19	1 ^K^	45.0%
13	LF + malaria	Nord	19	1 ^L^	28.0%
14	LF + malaria	Nord	19	1 ^M^	19.0%

^A^ = Anse à Veau, Arnaud, Asile, Fonds des Nègres, Grand Boucan, Miragoâne, Paillant, Petit Trou des Nippes, Petite Rivière des Nippes, Plaisance du Sud

^B^ = Saut d’Eau

^C^ = Saint Suzanne, Trou du Nord, Terrier Rouge

^D^ = Caracol

^E^ = Carice, Capotille, Ferrier, Fort Liberté, Ounaminthe, Mombin Crouchu, Mont Organise, Perches, Vallieres

^F^ = Chansolme

^G^ = Jean Rabel, Bombardopolis, Baie-de-Henne, Mole Saint Nicolas, Saint Louis du Nord, Bassin Bleu, Anse à Foleur

^H^ = Dondon

^I^ = Plaisance

^J^ = Limonade

^K^ = Plaine du Nord

^L^ = Cap Haitien

^M^ = Limbe

### Study design

The TAS is a school- or community-based survey which employs a sampling strategy (cluster, systematic or census) determined by the total number of children in the target age group (six and seven years old), number of clusters (schools or census enumeration areas), primary school enrollment rate, and vector and parasite species in predetermined EUs (Table 2). The TAS uses a critical cutoff for antigen prevalence in children, below which transmission is assumed to be no longer sustainable, even in the absence of MDA. When the number of LF positive cases among six and seven year olds is at or below the established threshold, the EU ‘passes’ the TAS and LF programs can decide to stop MDA. Surveys were designed using Survey Sample Builder (SSB) [19] with survey design for STH and malaria assessments the same as for TAS.

**Table 2:** Transmission Assessment Survey Design by Evaluation Unit.

EU number #	TAS- type	Number of Schools in EU	Number of Targeted Schools	Estimated number of Children Aged 6 & 7 Years in the EU	Target Sample Size	Critical Cutoff of LF positives	Survey Design	Sampling by Age or Grade	Assumed Absentee Rate
							C = Cluster; S = Systematic (Sampling Fraction, Interval)		
1	TAS	721	36	35,357	1,556	18	C (1.0, 1.0)	Grade	10%
2	TAS-STH-malaria	367	43	14,813	1,548	18	C (1.0, 1.0)	Grade	10%
3	TAS-malaria	67	38	2,442	1,228	14	C (1.0, 1.0)	Age	10%
4	TAS-malaria	120	30	6,821	1,524	18	C (1.0, 1.0)	Age	10%
5	TAS-malaria	17	17	707	365	4	S (0.57, 1.74)	Age	10%
6	TAS-malaria	333	31	18,977	1,552	18	C (1.0, 1.0)	Age	10%
7	TAS-malaria	25	25	1,597	530	6	S (0.39, 2.56)	Age	15%
8	TAS-malaria	441	39	20,883	1,552	18	C (1.0, 1.0)	Age	15%
9	TAS-malaria	26	29	754	365	4	S (0.57, 1.76)	Age	15%
10	TAS-malaria	34	34	1,679	594	7	S (0.42, 2.4)	Age	15%
11	TAS-malaria	42	42	1,336	780	9	C (0.96, 1.04)	Age	15%
12	TAS-malaria	48	31	1,634	891	11	C (1.0, 1.0)	Age	15%
13	TAS-malaria	199	39	9,299	1,532	18	C (1.0, 1.0)	Age	15%
14	TAS-malaria	74	30	4,038	1,380	16	C (1.0, 1.0)	Age	15%

### Survey teams

The LF-only TAS teams were composed of a total of four people: (i) facilitator (responsible for identifying and organizing eligible children); (ii) enroller (responsible for enrollment); (iii) laboratory technician; and (iv) reader (responsible for reading laboratory test and recording result).

Some of the TAS-malaria teams had an additional laboratory technician, for a total of 5 team members. The TAS-STH-malaria teams had two additional individuals to collect and process the stool specimens and to perform Kato Katz, for a total of six or seven team members. Team members received intensive training prior to the surveys.

### Population and school data

The target population for all three survey parts was children aged six and seven years. This age group is selected based on the assumption that this age group of children would have been born just before or during the annual MDA campaigns for LF, and therefore, they should not have been exposed to bites of mosquitoes carrying infective larvae.

Standard projection methods were used to estimate the population in the 14 EUs using data from Haiti’s most recent national census (2003).

Since comprehensive lists of school enrollment were not available from the Ministry of Education (MOE), trained community-level workers visited schools within the EU and survey areas to generate lists of all schools with the number and ages of children enrolled. The numbers of children (of the targeted ages) enrolled in school were compared with the projected population of children in the target age group for each EU to estimate percent school enrollment rates and to inform survey design.

### Sampling

Based on school enrollment rates, all 14 EUs were eligible to conduct school-based TAS [13]. The Haitian Ministry of Education indicated that 1^st^ and 2^nd^ grade students served as a reasonable proxy for six and seven year old children, therefore, selection criteria were set a priori to be conducted amongst 1^st^ and 2^nd^ grade students across the 14 EUs.

At each school, eligible children were selected to receive an LF test according to the sample interval prescribed by SSB (Table 2). In the EUs where TAS-malaria was conducted (2–14), all children selected for LF testing were also tested for malaria. In the 1 EU in which LF-malaria-STH TAS was conducted, an additional sampling interval was defined by SSB for selecting a subset of children to be tested for STH.

### Sample collection and field diagnostics (LF, STH and malaria)

All diagnostic tests for the integrated TAS were carried out in the field. The diagnostic tests used for LF, STH and malaria were the BinaxNOW Filariasis immunochromatographic test (ICT) (Alere, Maine), Kato Katz (Vestergaard-Frandsen, Denmark), and First Response Malaria Histidine-Rich Protein II (HRP2) (II3FRC30) (Premier Medical Corporation, New Jersey) rapid diagnostic test (RDT), respectively. Dried blood spots (DBS) were collected on calibrated filter paper (Cellabs, Australia) for subsequent serological testing.

From each enrolled child, technicians collected approximately 175 μL of blood from a single finger stick. Immediately following blood collection, 100 μL of blood was applied to the ICT and results were read at 10 minutes according to manufacturer instructions. Five μL of blood and 2 drops (60 μL) buffer were applied to the RDT and read at 20 minutes, according to manufacturer instructions. Sixty μl of blood was applied to calibrated filter paper and dried individually.

Serological assays are currently underway at CDC in Atlanta, GA, and results will be reported separately.

### Sample collection and field diagnostics (STH)

Stool cups were distributed at the time of enrollment. Stool samples were immediately processed on site and two slides were prepared and examined from each sample.

### Data collection and analysis

All enrollment information and diagnostic results were recorded directly into Blu® smart phones and uploaded to the cloud-based LINKS [20] server using software developed and supported by the NTD Support Center.

At the conclusion of the survey at each school visit, teachers and administration were given the diagnostic results.

Data were downloaded from the server in Microsoft Excel. They were cleaned, merged and analyzed using SAS 9.3 at CDC.

### Treatment

Children in whom any of the three diseases were detected were provided treatment according to national guidelines, using medications provided by the survey team. Those found to have LF or STH were given DEC+ALB or ALB, respectively. Children found to have malaria were referred to the nearest health public facility to receive chloroquine and primaquine treatment free of charge, in compliance with the MSPP’s antimalarial first-line drug recommendation for treating uncomplicated malaria.

## Results

Haiti’s NPELF and partners completed 14 TAS surveys between November 2014 and June 2015 (Fig 1 and Table 1). In all, 16,655 children were tested for LF, 14,795 for malaria, and 298 for STH (Table 3).

**Table 3:** Results: Transmission Assessment Survey.

EU #	# Schools Visited	Total Tested by ICT	% Female	% 6 Years	% 7 Years	# Positive ICT	# Negative ICT	# Positive RDT Malaria	# Negative RDT Malaria	TAS Pass/Fail
1	36	1,494	46.3%	19.0%	21.8%	0	1,494	-	-	PASS
2	45	1,662	44.7%	25.8%	22.3%	3	1,659	0	1,667	PASS
3	53	1,233	52.8%	38.8%	61.2%	2	1,231	7	1,191	PASS
4	45	1,528	50.0%	52.3%	47.7%	0	1,528	1	1,531	PASS
5	16	365	50.6%	56.0%	43.7%	1	364	0	365	PASS
6	42	1,619	48.8%	46.3%	53.7%	2	1,617	1	1,628	PASS
7	25	551	50.7%	45.1%	54.0%	0	551	0	545	PASS
8	47	1,589	55.9%	37.2%	62.8%	2	1,587	1	1,569	PASS
9	24	587	53.7%	47.0%	53.0%	0	587	0	585	PASS
10	30	672	50.7%	47.5%	52.5%	0	672	1	671	PASS
11	31	877	51.6%	41.2%	58.7%	19	858	1	856	FAIL
12	37	1,052	48.5%	38.0%	62.0%	15	1,037	0	1,073	FAIL
13	32	2,002	39.0%	33.8%	66.2%	18	1,984	1	2,004	MARGINAL
14	33	1,424	53.5%	43.0%	56.9%	10	1,414	0	1,417	PASS
**Total**	**460**	**16,655**	**49.1%**	**40.6%**	**53.8%**	**72**	**16,583**	**12**	**14,783**	** **

Haiti’s first TAS (LF only) was conducted in November 2014 in Sud Est department (EU 1). Teams visited 36 schools and found that zero (0%) of 1,494 children tested positive for LF by ICT.

The second TAS (TAS-STH-malaria) was conducted in February 2015 in Nippes department (EU 2). Teams visited 45 schools and found that 3 (0.2%) of 1,662 children tested positive for LF by ICT, 0 (0%) of 1,667 tested positive for malaria by RDT, and 46 (15.4%) of 298 children tested positive for STH (Kato Katz).

In the first two EUs, school grades poorly approximated age, with children enrolled in first and second grades ranging in age from five to 18 years. Despite testing children as old as 18 years of age, who could be more likely to have contracted LF before the start of MDA campaigns, only 3 children tested positive for LF in EU 2. In subsequent EUs, only six and seven year old children (from any grade) were identified and eligible for testing.

From February to June 2015, TAS-malaria surveys were carried out in Nord, Nord Est, Nord Ouest and Centre departments (EUs 3–14). In total, teams visited 379 schools and tested more than 13,000 children for LF and malaria. In all, nine EUs passed the TAS. One (EU 13) marginally passed TAS with 18 positive for LF, which met, but did not surpass the critical threshold for continuation of LF MDA. Two EUs (11 and 12) failed the TAS with 19 and 15 children testing positive for LF antigen by ICT, surpassing their respective thresholds of 9 and 11, respectively. Twelve of 13,128 children tested had positive RDT results.

### Estimated survey costs

Team members spent a total of 316 working days (working day = 1 person working for 1 day) in the field to accomplish the TAS in 14 EUs. The LF-only TAS, the TAS-malaria and the TAS-STH-malaria required an average of 22, 22.5, and 27 working days per EU, respectively. In terms of productivity, LF-only TAS, TAS-malaria and TAS-STH-malaria teams completed activities at the same rate of 1.6 schools/day, accomplished by the additional personnel for the integrated TAS surveys. The cost of the TAS-malaria evaluation was an estimated 15% higher than the cost for the LF-only evaluation. The cost of the TAS-STH-malaria evaluation was 49% higher than the cost for the LF-only evaluation. The additional costs resulted from resources required for additional personnel and their transportation.

## Discussion

Integrated surveys have the potential to both optimize resource utilization (human and financial) and generate useful data across programs using a robust survey platform. The Integrated TAS-STH-malaria survey was found to be feasible and generated useful information for all three programs.

Twelve of the 14 EUs passed the LF TAS, allowing the program to stop MDA for LF in 12 EUs, or 45 communes. In 36 of the communes that were able to stop MDA, baseline LF prevalence was low; however, the NPELF also succeeded in reducing LF prevalence sufficiently to pass TAS in 8 communes where LF prevalence was moderate or high at the time of mapping in 2000–2001. This achievement underscores the quality of the MDA activities and the ability of the program to achieve adequate participation of the population. In the two EUs that failed TAS (EU 12, 13), MSPP and partners will continue MDA for an additional two rounds, before re-evaluating in sentinel and spot check sites. Though EU 13 technically passed the TAS with 18 children positive out of 2,002 tested, the EU is surrounded by other areas of ongoing transmission, and thus MSPP and partners took the conservative decision to continue MDA there for an additional two rounds.

According to the WHO TAS-STH manual, the 46 positive STH results from EU 2 would categorize the area as having a prevalence range of 10% to <20% [14]. This represents a decrease in STH prevalence from the 2002 national survey, but leads to the programmatic recommendation to conduct annual, school-based treatment for STH in that EU to maintain reduced STH levels.

In all, malaria was detected by RDT in only 12 of 14,795 children in 38 communes. The inclusion of malaria RDTs in the LF TAS confirms that malaria prevalence in Haiti is low, and provides additional evidence in support of the decision to continue current programs and the development of new strategies in surveillance toward malaria elimination. Although few malaria RDT-positive individuals were identified amongst a limited population, antibody assays should provide a cumulative history of exposure and potentially define transmission foci for both diseases.

The integrated TAS strategy has several advantages. Most importantly, the integrated TAS-STH-malaria enabled partners to make programmatic decisions for stopping LF MDA and for deworming frequency for STH. As the LF program nears elimination, new approaches to STH monitoring and control, including the integrated TAS which produces actionable results for STH, should be incorporated into work plans.

Second, the integrated TAS establishes a potential framework for integrated surveillance and a more coordinated approach to community-based intervention strategies. By testing for multiple infections, we were able to generate actionable results for more than one program. The results also informed thinking about the testing needed to support malaria elimination. In a single, integrated and carefully planned survey, we are able to maximize returns on the investment of field activities while also reducing burden on both field teams and communities. These synergies are especially important for diseases in the elimination phase, since surveillance efforts will be resource intensive for increasingly rare conditions.

Third, this activity fostered collaboration between ministries of education (MOE) and health (MSPP), and across disease programs within MSPP. The integrated TAS marks the first time in which field activities for the three disease programs were combined in such a manner in Haiti. The NPELF and partners conveyed the common goals of each public health activity to encourage school administrators to allow field teams to conduct integrated TAS in schools. The support of school administrators will be particularly important as STH control moves towards school-based deworming following the end of LF MDA. The LF, malaria and STH programs and field teams worked well together in this mutually beneficial and informative activity, thereby developing and strengthening the new working relationship.

Fourth, the integrated survey represented an overall cost savings, compared with performing similar assessments independently. The addition of the malaria assessment to the TAS (TAS-malaria) cost an estimated 15% more in Haiti than the LF-only TAS, due to the addition of a team member for processing and reading of malaria RDTs. As teams become more familiar with the workflow, it is anticipated an additional team member might not be necessary to perform the malaria RDT. The integrated TAS-STH-malaria costs an estimated 49% more than the LF-only TAS, due to the addition of two team members for processing of stool by Kato Katz. Obtaining the same information through separate surveys conducted by each independent program would have incurred substantially more expense. The integrated survey platform creates options to include additional tests to expand the utility of the survey.

Lastly, the integrated TAS represented less of an intrusion for the communities than would three independent surveys. By performing multiple diagnostic tests in one coordinated visit, the amount of time that children were kept from classes was minimized. The overall activity was less time consuming, in that the school administration was approached only once on behalf of the three programs, and consent was obtained for all diagnostics concurrently. The one visit proved to take only slightly longer than the TAS-only activity, and provided immediate results to communities and treatment for infected children.

This study identified a few challenges to performing the TAS in Haiti. First, the age of the children enrolled in first and second grades varies greatly, so the program was unable to rely on grades as a proxy for age. Testing children older than seven years (i.e. born prior to the start of MDA), provides a more conservative estimate of LF transmission since it includes children were born before MDA began, however it fails to answer the question about the effect of MDA on recent transmission. For this reason, the decision was made to identify children by age in EUs 3–14, rather than continuing to use grade as a proxy for age in order to better keep with the TAS protocol guidelines.

Second, the estimate of student enrollment frequently exceeded actual child attendance on the day of the surveys. More schools needed to be visited in order to reach the predetermined sample size, which posed additional logistical burden on the field teams. The TAS survey guidelines rely on accurate estimations of the number of children of the target ages living in each EU as well as school enrollment rates, to ensure that survey design yields representative and appropriately distributed samples from across the targeted population. Prior to the survey, current school enrollment rates were not available from the MOE, so partners obtained school data directly from the schools in each EU.

Third, field teams had to manage several logistical challenges including limited access to cold chain for storage of ICT cards before TAS and dried blood spots after TAS. Limited cellular service, accessibility and difficult terrain of some selected schools, and school holidays were a challenge for field teams impacting sample size. Finally, sensitization was not always sufficient, leading to refusals in some instances.

There were also limitations associated with the malaria program. First, although testing more than 14,700 six and seven year old children confirmed the low prevalence of malaria in the surveyed areas, the rates of RDT positivity was low and consequently, the results of the malaria antibody testing planned for specimens collected during this study are likely to be more informative for programmatic decisions than the RDT results. Second, the integrated TAS survey design, including geographic distribution of EUs, was based on historical LF mapping data. Use of malaria distribution as the basis for the creating EUs would have likely influenced the choice of how to combine communes to form EUs.

## Conclusions

The activities reported here from 14 TAS surveys provide evidence of the feasibility of using the LF TAS as a platform for integration of assessments for STH and or malaria.

In 2014, Haiti’s LF elimination program achieved eligibility for administration of TAS in 47 communes and after conducting TAS was able to stop MDA campaigns for approximately 1,981,920 people in 44 communes. Intensified activities in the next five years, including progressive implementation of TAS, stopping MDA, and phasing in post-MDA surveillance, will be essential to achieving the country’s elimination goal by 2020 [21]. With this in mind, the NPELF and partners plan to conduct TAS (including TAS-STH-malaria and TAS-malaria) in 58 additional communes in 2016 and 39 additional communes before the end of 2017. Integrating assessment of STH infections will enable program managers to determine the effectiveness of proposed school-based STH programs, while Integrating malaria into the TAS platform will provide additional national data on recent malaria history, which is important for targeting malaria elimination efforts and ensuring progress towards a malaria-free Haiti. Despite experiencing many challenges, including the 2010 earthquake and cholera outbreak, integrated TAS results support the assertion that Haiti is on track to meeting the WHO’s 2020 LF global elimination targets. Although malaria elimination is admittedly a more ambitious goal, development of integrated surveillance strategies will help to achieve this goal.

## References

[1] World Health Organization (2012) Global programme to eliminate lymphatic filariasis: progress report for 2011. Weekly epidemiological record 87: 345–356. 22977952

[2] World Health Organization (2013) Global programme to eliminate lymphatic filariasis: progress report for 2012. Weekly epidemiological record 88: 389–400. 24073461

[3] Hooper PJ, Chu BK, Mikhailov A, Ottesen EA, Bradley M. (2014) Assessing Progress in Reducing the At-Risk Population after 13 Years of the Global Programme to Eliminate Lymphatic Filariasis. PLoS Negl Trop Dis. 8: e3333 10.1371/journal.pntd.0003333 25411843 PMC4239000

[4] WHO (2012) Soil Transmitted Helminthiases: Eliminating Soil Transmitted Helminthiases as a Public Health Problem in Children–Progress Report 2001–2010 and Strategic Plan 2011–2020. Geneva: World Health Organization, 2012.

[5] World Health Organization (2015) Soil Transmitted Helminthiases: number of children treated in 2014. Weekly epidemiological record 90: 701–712.

[6] World Health Organization (2015) Global Malaria Programme: World Malaria Report 2015. Geneva: World Health Organization, 2015.

[7] WHO. Global Technical Strategy for Malaria 2016–2030. Geneva: World Health Organization, 2015.

[8] Raccurt C. Le point sur le paludisme en Haïti (in French). Santé. 2004; 14:201–4. 15745868

[9] Beau de Rochars ME, Milord MD, Yvan SJ, Desormeaux AM, Dorvil JJ, Lafontant JG, Addiss DG, Streit T. Geographic distribution of lymphatic filariasis in Haiti. American J Trop Med Hyg. 2004; 71(5): 598–601. 15569791

[10] Lemoine JF, Desormeaux AM, Monestime F, Fayette CR, Desir L, Direny AN, Craciunoiu S, Miller L, Knipes A, Lammie P, Smith P, Stockton M, Trofimovich L, Bhandari K, Reighinger R, Crowley K, Ottesen E, Baker M. (2016) Controlling Neglected Tropical Diseases (NTDs) in Haiti: Implementation Strategies and Evidence of Their Success. PLoS Negl Trop Dis 10(10): e0004954 10.1371/journal.pntd.0004954 27706162 PMC5051938

[11] Lucchi NW, Karell MA, Journel I, Rogier E, Goldman I, Ljolje D, Huber C, Mace KE, Jean SE, Akom EE, Oscar R, Buteau J, Boncy J, Barnwell JW, Udhayakumar V. PET-PCR method for the molecular detection of malaria parasites in a national malaria surveillance study in Haiti, 2011. Malar J. 2014; 11 26; 13: 462 10.1186/1475-2875-13-462 25428550 PMC4289323

[12] Lindblade KA, Steinhardt L, Samuels A, Kachur SP, Slutsker L. The silent threat: asymptomatic parasitemia and malaria transmission. Expert Rev Anti Infect Ther. 2013; 6 11(6): 623–39. 10.1586/eri.13.45 23750733

[13] Elbadry MA, Al-Khedery B, Tagliamonte MS, Yowell CA, Raccurt CP, Existe A, Boncy J, Weppelmann TA, Beau De Rochars VEM, Lemoine JF, Okech BA, Dame JB. High Prevalence of Asymptomatic Malaria Infections: A Cross-Sectional Study in Rural Areas in Six Departments in Haiti. Malar J. 2015; 14: 510 10.1186/s12936-015-1051-2 26689195 PMC4687167

[14] WHO. Global Programme to Eliminate Lymphatic Filariasis: Monitoring and epidemiological assessment of mass drug administration: a manual for national elimination programs. Geneva: World Health Organization, 2011.

[15] WHO. Manual: Assessing the Epidemiology of STH during a TAS. Geneva: World Health Organization, 2015.

[16] Chu BK, Gass K, Batcho W, Ake M, Dorkenoo AM, Adjinacou E, Mafi E, Addiss DG. Pilot Assessment of Soil-Transmitted Helminthiasis in the Context of Transmission Assessment Surveys for Lymphatic Filariasis in Benin and Tonga. PLoS Negl Trop Dis. 2014; 8(2)e2708 Epub 2014/02/20. 10.1371/journal.pntd.0002708 24551267 PMC3923741

[17] Drabo F, Ouedraogo H, Bougma R, Bougouma C, Bamba I, Zongo D, Bagayan M, Barrett L, Yago-Wienne F, Palmer S, Chu B, Toubali E, Zhang Y. (2016) Successful Control of Soil-Transmitted Helminthiasis in School Age Children in Burkina Faso and an Example of Community-Based Assessment via Lymphatic Filariasis Transmission Assessment Survey. PLoS Negl Trop Dis 10(5): e0004707 10.1371/journal.pntd.0004707 27163294 PMC4862685

[18] Gunawardena S, Gunawardena NK, Kahathuduwa G, Karunaweera ND, de Silva NR, Ranasinghe UB, Samarasekara SD, Nagodavithana KC, Rao RU, Rebollo MP, Weil GJ. Integrated School-based Surveillance for Soil-Transmitted Helminth Infections and Lymphatic Filariasis in Gampaha District, Sri Lanka. Am J Trop Med Hyg. 2014; 90(4):662–6. Epub 2014/02/05. 10.4269/ajtmh.13-0641 PMC3973510 24493672

[19] Task Force for Global Health. TAS STH Survey Sample Builder, version 1.51. Available: http://www.ntdsupport.org/resources/tas-sth-survey-sample-builder-tool. Accessed 28 Feb 2016.

[20] Pavluck A, Chu B, Mann Flueckiger R, Ottesen E. Electronic Data Capture Tools for Global Health Programs: Evolution of LINKS, an Android-, Web-Based System. PLoS Negl Trop Dis. 2014; 8(4): e2654 10.1371/journal.pntd.0002654 24722343 PMC3983089

[21] Ichimori K, King JD, Engels D, Yajima A, Mikhailov A, Lammie P, Ottesen EA. Global Programme to Eliminate Lymphatic Filariasis: The Processes Underlying Programme Success. PLoS Negl Trop Dis. 2014; 8(12): e3328 10.1371/journal.pntd.0003328 25502758 PMC4263400

